# Probing the active site of Class 3 L-asparaginase by mutagenesis. I. Tinkering with the zinc coordination site of ReAV

**DOI:** 10.3389/fchem.2024.1381032

**Published:** 2024-04-04

**Authors:** Kinga Pokrywka, Marta Grzechowiak, Joanna Sliwiak, Paulina Worsztynowicz, Joanna I. Loch, Milosz Ruszkowski, Miroslaw Gilski, Mariusz Jaskolski

**Affiliations:** ^1^ Institute of Bioorganic Chemistry, Polish Academy of Sciences, Poznan, Poland; ^2^ Department of Crystal Chemistry and Crystal Physics, Faculty of Chemistry, Jagiellonian University, Cracow, Poland; ^3^ Department of Crystallography, Faculty of Chemistry, Adam Mickiewicz University, Poznan, Poland

**Keywords:** hydrolase, amidohydrolase, L-asparaginase, leukemia, metalloprotein, site-directed mutagenesis, ITC, X-ray crystallography

## Abstract

ReAV, the inducible Class-3 L-asparaginase from the nitrogen-fixing symbiotic bacterium *Rhizobium etli*, is an interesting candidate for optimizing its enzymatic potential for antileukemic applications. Since it has no structural similarity to known enzymes with this activity, it may offer completely new ways of approach. Also, as an unrelated protein, it would evade the immunological response elicited by other asparaginases. The crystal structure of ReAV revealed a uniquely assembled protein homodimer with a highly specific C135/K138/C189 zinc binding site in each subunit. It was also shown before that the Zn^2+^ cation at low and optimal concentration boosts the ReAV activity and improves substrate specificity, which indicates its role in substrate recognition. However, the detailed catalytic mechanism of ReAV is still unknown. In this work, we have applied site-directed mutagenesis coupled with enzymatic assays and X-ray structural analysis to elucidate the role of the residues in the zinc coordination sphere in catalysis. Almost all of the seven ReAV muteins created in this campaign lost the ability to hydrolyze L-asparagine, confirming our predictions about the significance of the selected residues in substrate hydrolysis. We were able to crystallize five of the ReAV mutants and solve their crystal structures, revealing some intriguing changes in the active site area as a result of the mutations. With alanine substitutions of Cys135 or Cys189, the zinc coordination site fell apart and the mutants were unable to bind the Zn^2+^ cation. Moreover, the absence of Lys138 induced atomic shifts and conformational changes of the neighboring residues from two active-site Ser-Lys tandems. Ser48 from one of the tandems, which is hypothesized to be the catalytic nucleophile, usually changes its hydration pattern in response to the mutations. Taken together, the results provide many useful clues about the catalytic mechanism of the enzyme, allowing one to cautiously postulate a possible enzymatic scenario.

## Highlights

• The prototypic *Rhizobium etli* Class 3 ReAV enzyme is a new addition to the growing L-asparaginase superfamily, with an active site, comprised of two Ser-Lys tandems and a nearby 2xCys-Lys zinc coordination center, tentatively identified by structural homology with other enzymes and by pan-genomic sequence analyses.
• We test the predictions by site-directed mutagenesis around the zinc cation, followed by enzymatic assays and X-ray crystal structure determinations.
• Ala mutations of the coordinating Cys residues render the enzyme inactive. The K138A mutation in the coordination site impairs zinc binding and decreases the K_M_ to submillimolar level, making the protein interesting for optimization as an antileukemic drug.

## 1 Introduction

Asparaginases (EC 3.5.1.1), which split L-asparagine to L-aspartate and ammonia, are grouped into three unrelated structural classes: Class 1 (bacterial-type), Class 2 (plant-type), and Class 3 (*Rhizobium etli*-type) (Loch and Jaskolski, 2021; da Silva et al., 2022). Due to terminological complications, this structure-based classification of L-asparaginases has been recently proposed to replace the older classification according to the source organism of the first enzymes discovered. The historical division is no longer correct because all three groups of L-asparaginases are distributed in all domains of life. Class 1 L-asparaginases can be subdivided into two types: cytosolic type I enzymes expressed constitutively, and periplasmic type II enzymes induced under anaerobic conditions (Campbell et al., 1967). Class 2 (type III) L-asparaginases belong to the family of N-terminal nucleophile (Ntn) amidohydrolases, and can be found not only in plants but also in microbes and mammals (Borek and Jaskolski, 2001; Michalska et al., 2005). L-Asparaginases of Class 3 were originally found in and named after *R. etli*, although they have a much wider appearance in bacteria (Zielezinski et al., 2022), and are also found in fungi (Loch and Jaskolski, 2021).

L-Asparaginase is the cornerstone of the clinical treatment of acute lymphoblastic leukemia (ALL). The availability of circulating L-asparagine is a crucial factor for the survival of the malignant lymphoblasts, as they have low expression level of asparagine synthetase and, therefore, are unable to produce enough asparagine needed for their rapid growth (Abaji and Krajinovic, 2019). By clearing L-asparagine from circulation, L-asparaginases starve the asparagine-dependent cancer cells, but not the healthy cells, to death (Radadiya et al., 2020). Some Class 1 (bacterial-type II) L-asparaginases have been used in clinical ALL chemotherapy, including the *Escherichia coli* enzyme EcAII (Elspar) (Swain et al., 1993), its PEGylated version (Oncaspar) (Dinndorf et al., 2007) and an alternative bacterial enzyme from *Erwinia chrysanthemi* ErAII (Erwinase) (Gervais et al., 2013). At present, only periplasmic L-asparaginases from *E. coli* and *E. chrysanthemi*, with sufficiently low K_M_, have been proven to be effective antileukemics, although their administration is often associated with serious side effects (Medawar et al., 2020). Alternative sources of therapeutic asparaginases have been thus sought, and Class 3 L-asparaginases emerge as interesting candidates.

*R. etli* is a soil-dwelling bacterium capable of forming a symbiotic relationship with legumes, such as *Phaseolus vulgaris* (common bean), in which they fix atmospheric nitrogen into the chemical form of ammonia. The bacteria symbionts benefit from plant carbon compounds as a source of the energy needed for nitrogen fixation (Huerta-Zepeda et al., 1996). *R. etli* has two asparaginases, differentiated by their thermostability and electrophoretic mobility. The constitutive ReAIV enzyme (*R. etli* type IV in Class 3) is thermostable and migrates faster in native electrophoretic gels, while the thermolabile and inducible ReAV enzyme (*R. etli* type V in Class 3) is positively regulated by the L-asparagine substrate (Huerta-Zepeda et al., 1997). The sequences of these two proteins show a low level of identity (∼30%) and both are significantly different from the sequences of Class 1 and Class 2 asparaginases (Loch and Jaskolski, 2021). The recently reported crystal structure of ReAIV (Loch et al., 2023) showed that despite the low sequence identity, the two enzymes have basically the same 3D structure.

**FIGURE 1:**
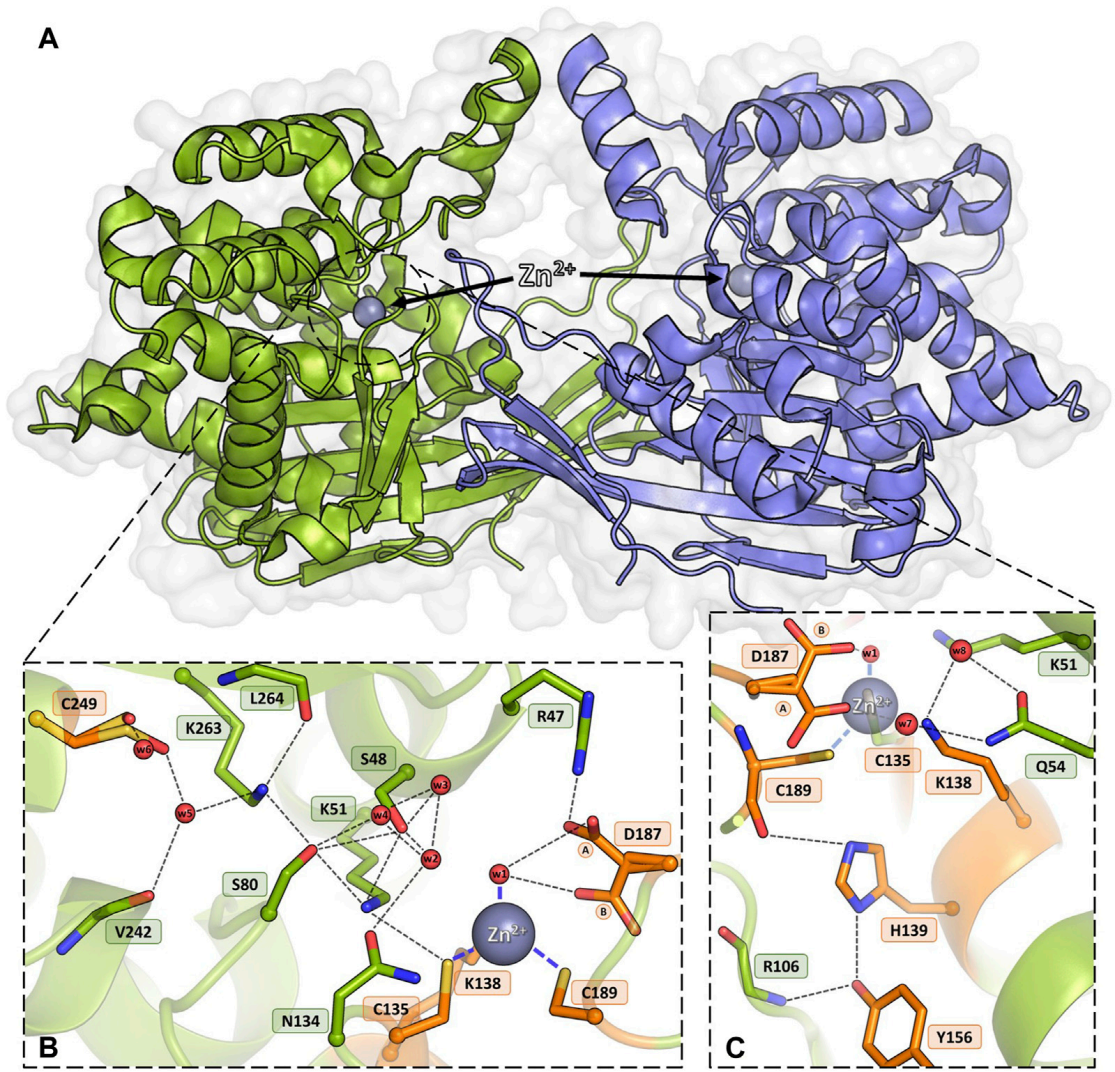
Homodimeric ReAV protein with the active sites containing Zn^2+^ cations (gray spheres). **(A)** An overview of the dimeric assembly of ReAV with the active site located in a cleft on the protomer surface. **(B)** Detailed view of the active site area of ReAV, with the highly specific C135/K138/C189 zinc binding site and the most important hydrogen bond connections (thin dash lines). **(C)** Interactions of the highly conserved residues H139 and Y156, located in the close vicinity of the metal cation. The alternative conformations of Asp187 are indicated by circled capital letters A and B. Water molecules are shown as small red spheres. The residues mutated in this work are colored and labeled in orange.

The structural canon of Class 3 L-asparaginases was established by the crystal structure of the ReAV enzyme (Loch et al., 2021), which showed a protein with no similarity to Class 1 or 2 members, but with curious resemblance to some serine β-lactamases, Penicillin Binding Proteins (PBPs), and glutaminases. As the ReAV enzyme shows no sequence or structure similarity to other asparaginases, it is likely to function according to a new catalytic mechanism. ReAV is a homodimer with an α/β protomeric fold, formed by two tightly packed domains (Figure 1A). The active site of the enzyme is located in a cleft on the protomer surface and marked by a zinc ion with an unusual coordination sphere created by Cys135, Lys138, Cys189, and a water molecule (Figure 1B). The conspicuous catalytic center of the enzyme comprises two Ser-Lys tandems (Ser48-Lys51 and Ser80-Lys263), centered around the curiously hydrated Ser48 residue and located in the close vicinity of the metal binding site. It was established that Zn^2+^ binding is highly selective (Loch et al., 2021), with K_D_ of ∼3 μM. Moreover, it was shown that low and optimal zinc concentration can boost the activity of ReAV by over 50%, while simultaneously improving the substrate specificity, indicating its role in substrate recognition (Sliwiak et al., 2024). There is also an indirect connection between Cys135 from the metal coordination sphere and an oxidized Cys249, which is involved in a network of H-bonds comprising the entire active site area (Figure 1B). The oxidizable Cys249 residue may play a role in protection of the zinc binding site against reactive oxygen species (ROS) (Loch et al., 2021).

**SCHEME 1:**
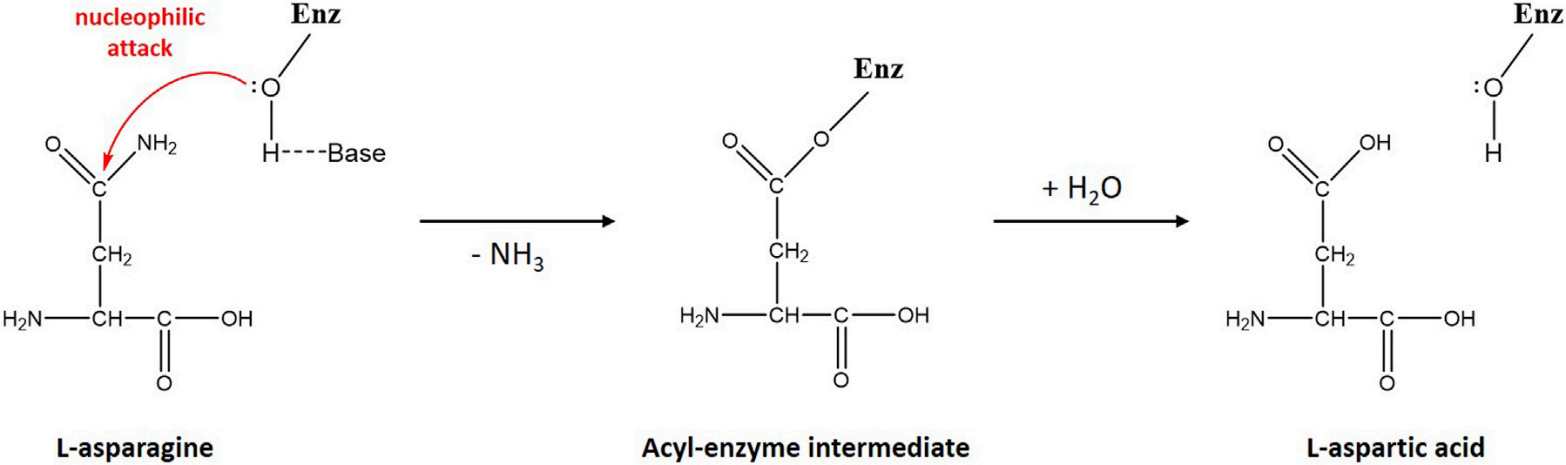
Schematic diagram of L-asparagine hydrolysis by ReAV. The primary nucleophile, activated by a nearby general base, is the hydroxyl group of Ser48. The first step of the reaction leads to the creation of a β-acyl-enzyme, with the release of ammonia. In the second step, a water molecule hydrolyzes the ester bond of the β-acyl-enzyme, leading to the release of the second product (L-Asp) and recreation of the original state of the enzyme.

A DALI (Holm et al., 2023) search of the PDB database revealed some structural homologs of ReAV belonging to unrelated protein families. The homologs were mostly glutaminases (rmsd 2.9–3.4 Å), e.g., from *Micrococcus luteus* (PDB ID 3ih8) or *E. coli* (1u60), and serine β-lactamases (rmsd 2.8–3.7 Å), e.g., from *Acinetobacter baumanii* (5l2f) or *Pseudomonas aeruginosa* (5eoo). Sequence similarity between ReAV and the above homologs is, however, very low and does not exceed 15%. The highest degree of structure similarity is seen in the region of the catalytic domain (Loch et al., 2021). The putative active site of ReAV resembles the arrangement of the catalytic residues of the structural homologs in the area encompassing the two serine-lysine tandems (Ser48-Lys51 and Ser80-Lys263), with the hydrated Ser48 identified as the catalytic nucleophile. On the other hand, one of the most striking differences is the absence of a metal cation in the active site of the structural homologs. The zinc coordination site is, therefore, a unique feature of the ReAV enzyme. However, the metal-binding region is preserved in the structurally homologous proteins and is usually occupied by an amino acid side chain. As another difference, the structural homologs of ReAV have different and variable oligomeric architecture. A hypothetical catalytic mechanism of ReAV, assuming a two-step reaction with an acyl-enzyme intermediate, is presented in Scheme 1.

A recent pan-genomic analysis of L-asparaginase distribution was aimed *inter alia* at the identification of ReAV orthologs within the bacterial kingdom (Zielezinski et al., 2022). Screening of over 45,000 bacterial genomes revealed that *Rhizobium etli*-type enzymes are present far beyond the *Rhizobium* genus. Moreover, the ReAV protein shows the highest sequence similarity to proteins found in the distantly related *Burkholderia* species, strongly suggesting a horizontal gene transfer (HGT) from *Burkholderia*. The identification of sequence homologs of ReAV, including ReAIV, allowed us to trace the conservation pattern of the key residues, including those in the active site of the enzyme and in the metal coordination sphere.

While wild-type (WT) ReAV is a fast enzyme (K_M_ 2.1 mM, k_cat_ 603 s^−1^, k_cat_/K_M_ 294 mM^−1^·s^−1^ at pH 9.0) (Sliwiak et al., 2024), its substrate affinity is by ca. two orders of magnitude too low for application as an antileukemic drug. However, the uniqueness of the ReAV sequence, and thus the possibility of a different catalytic mechanism, opens up new avenues for structure-based enzyme engineering focused on creating variants with improved catalytic properties. In this context, it is of note that the Class 2 *E. coli* EcAIII asparaginase has been the subject of a mutational campaign aimed at creating a more efficient enzyme (Loch et al., 2022; Janicki et al., 2023; Loch et al., 2024).

The exceptional architecture and properties of the inducible *R. etli* ReAV L-asparaginase make it an excellent candidate for mutagenetic experiments to probe the catalytic mechanism and improve its substrate affinity. Extensive analysis of available bacterial genomes as well as comparisons with structural homologs in the PDB allowed us to identify the most conserved regions of the ReAV sequence, including the residues involved in zinc coordination (Cys135, Lys138, Cys189), and residues located in the vicinity of the metal binding site (His139, Tyr156, Asp187, Cys249). The main goal of the present work is to assess the structural and catalytic role of the predicted residues by site-directed mutagenesis, coupled with crystal structure determination and enzymatic assays.

## 2 Methods

### 2.1 Site-directed mutagenesis

Site-directed mutagenesis of ReAV was performed using the polymerase incomplete primer extension (PIPE) technique (Klock and Lesley, 2009) (K138A, C189A, C249A) or according to Q5-site mutagenesis protocol (C135A, H139A, Y156A, D187A) to introduce alanine substitutions at designated positions. The mutations were introduced using specific primers, as listed in Table 1. The pET151-D-ReAV plasmid carrying the original protein sequence was used as template for PCR amplification. To eliminate transformation background, template DNA was digested with DpnI restriction enzyme according to the manufacturer’s protocol. The reaction products were used for transformation of *E. coli* BL21-Gold (DE3) competent cells (Agilent Technologies). After overnight incubation on LB agar plates supplemented with 100 μg∙mL^−1^ ampicillin, single colonies were inoculated into 4 mL of LB medium containing ampicillin, and grown overnight at 37°C. Following overnight incubation, plasmids were isolated and sequenced to confirm the presence of the desired mutations by Sanger sequencing (Genomed, Poland).

**TABLE 1:** Sequences of mutagenic primers with mutation sites underlined.

Mutant (A)	Forward primer	Reverse primer
C135	TTG​CAG​CAA​TGCCTCG​GGC​AAG​C	ACCGCCGTCGGGATAAAG
K138	TGC​TCG​GGCGCGC​ACG​TCG​GCA​TGC​TTG​CC	CCG​ACG​TGC​GCG​CCC​GAG​CAA​TTG​CTG​CAA​ACC
H139	TCG​GGC​AAGGCCGTC​GGC​ATG​CTT​GCC​GG	ATG​CCG​ACG​GCC​TTG​CCC​GAG​CAA​TTG​CTG​CAA​A
Y156	ACG​GAT​GGCGCCCAC​CTG​CCG​GAT​CAT​CCG	GGC​AGG​TGG​GCG​CCA​TCC​GTG​CCG​GCT​CC
D187	TGG​GGA​ACCGCCGGA​TGC​AAT​CTC​CCG​ACC​CC	ATT​GCA​TCC​GGC​GGT​TCC​CCA​TTC​GAC​GTC​CC
C189	ACC​GAC​GGAGCCAAT​CTC​CCG​ACC​CCC​GC	GGG​AGA​TTG​GCT​CCG​TCG​GTT​CCC​CAT​TCG​AC
C249	GGA​CGT​TACGCCACG​ATG​CTG​ATG​CGC​GC	AGC​ATC​GTG​GCG​TAA​CGT​CCC​TCG​CCC​GC

### 2.2 Protein expression and purification

Successfully sequenced plasmids were used for large-scale protein production. Competent bacterial cells (*E. coli* BL21 Gold) were transformed with expression constructs and the transformants were inoculated into 10 mL of LB medium containing 100 μg∙mL^−1^ ampicillin. After overnight incubation at 37°C, the preculture was transferred to 1 L of LB medium supplemented with ampicillin and cultured at 37°C until the OD_600_ reached ∼0.7. The temperature was then lowered to 18°C and protein expression was induced with 0.2 mM isopropyl β-D-thiogalactopyranoside (IPTG). After overnight induction, cells were harvested by centrifugation at 5,000 rpm for 10 min at 4°C and the cell pellet was re-suspended in 35 mL of binding buffer (50 mM Tris-HCl pH 8.0, 500 mM NaCl, 20 mM imidazole, 10% v/v glycerol, 1 mM tris(2-carboxyethyl)phosphine (TCEP)). The cells were frozen and kept at −80°C. After thawing, cells were disrupted by sonication and pelleted by centrifugation at 16,000 rpm for 30 min at 4°C. Clarified cell lysate was loaded on affinity column filled with HisTrap HP resin and equilibrated with binding buffer. The protein was eluted with elution buffer (50 mM Tris-HCl pH 8.0, 500 mM NaCl, 300 mM imidazole, 10% v/v glycerol, 1 mM TCEP) and dialyzed overnight with Tobacco Etch Virus (TEV) protease at 4°C against 50 mM Tris-HCl buffer pH 8.0, containing 500 mM NaCl and 1 mM TCEP. The digested protein sample was applied again on a HisTrap column to remove His-tag debris and the His-tagged TEV protease, and the flow-through containing the recombinant ReAV protein was collected. Protein sample was concentrated and loaded on HiLoad 16/60 Superdex 200 column equilibrated with 50 mM Tris-HCl buffer pH 8.0, containing 150 mM NaCl and 1 mM TCEP. Protein fractions were collected and sample purity was analyzed by SDS-PAGE. Fractions containing pure protein were pooled and concentrated using Amicon Ultra-15 (30000 MWCO) centrifuge filters.

### 2.3 Determination of specific activity, zinc profiles, kinetic parameters and zinc affinities of ReAV mutants

In order to find the optimal zinc concentration for WT ReAV and its mutants, L-asparaginase activity was determined by the Nessler method using the protocol from Loch et al. (2023) with 10 mM L-Asn in 20 mM Tris-HCl buffer pH 9.0 and enzyme concentration in the range of 60 nM–3.8 µM, in the presence of zinc ions in concentration varied from 1 to 100 μM (as was the case of the WT and K138A proteins), and additionally extended to the nanomolar range (150–500 nM) in the case of the H139A mutant. The relative activity expressed as the percentage of enzyme activity without zinc supplementation was calculated and plotted to compare the shape of the zinc profiles and the level of response to Zn^2+^. The kinetic parameters without and with optimal zinc concentration in 10 mM Tris-HCl pH 9.0 buffer were determined by the multiple injection isothermal titration calorimetry method (ITC-MIM), as described in Sliwiak et al. (2024). Briefly, the average enthalpy of total conversion of L-Asn was determined by four injections of 2 µL of 10 mM L-Asn (Sigma-Aldrich) into the reaction cell containing 2–10 µM of the enzyme, using 150–600 s intervals. In a blank experiment, L-Asn was injected into the reaction cell containing the buffer only. Next, heat-rate shift experiment was performed by injecting L-Asn at 100 mM concentration (in the syringe) in 20 aliquots of 1.8 µL, with 60 s intervals, into the reaction cell with the enzyme kept at 5–10 µM concentration, depending on sample activity. All measurements were taken at 37°C, with stirring at 700 rpm and differential power set to 10 μcal·s^−1^. Raw rate data were analyzed using the Enzyme Kinetics – Multiple Injections fitting model implemented in the MicroCal PEAQ-ITC Analysis Software (Malvern). Final kinetic parameters were calculated by averaging the values obtained from two separate experiments.

In order to assess the ability of the muteins to bind Zn^2+^ as well as their saturation with this ion, titrations (without any protein pretreatment with a chelator) with Zn^2+^ were performed using a MicroCal iTC200 calorimeter (GE Healthcare) as follows. Protein in the cell at ∼120–150 µM concentration (determined by UV absorption at 280 nm) was titrated with 2 µL aliquots of 1.3–2.0 mM ZnCl_2_. Both, ZnCl_2_ and the protein were in 25 mM Tris-HCl buffer pH 8.0, with 100 mM NaCl and 1 mM TCEP. The raw ITC data were analyzed using MicroCal PEAQ-ITC Analysis Software (Malvern) to obtain the following thermodynamic parameters of the complexation reactions: stoichiometry (N), dissociation constant (K_D_), as well as changes in enthalpy (ΔH) and entropy (ΔS). The measurements were performed in duplicate.

### 2.4 Crystallization

Prior to crystallization, ReAV mutant proteins were concentrated to 14–17 mg·mL^−1^ and supplemented with n-dodecyl-β-D-maltoside (DDM) to its final concentration of 0.5%–1% v/v. Vapor diffusion crystallization screening was based on the crystallization conditions for the WT protein (Loch et al., 2021). Crystals were grown at 19°C within 2–5 days from the crystallization solutions listed in Table 2. Crystals were cryoprotected in their mother liquor supplemented with ethylene glycol, vitrified in liquid nitrogen, and stored until X-ray data collection.

**TABLE 2:** Crystallization conditions for ReAV mutants.

Mutant (A)	Crystal form	Protein concentration	Crystallization solution
C135	orthorhombic	16 mg∙mL^−1^	30% PEG 4000, 0.2 M Li_2_SO_4_, 0.1 M Tris-HCl pH 8.5
K138	monoclinic	20 mg∙mL^−1^	22% PEG 3350, 0.2 M Li_2_SO_4_, 0.1 M Bicine pH 9.0
H139	monoclinic	13 mg∙mL^−1^	22% PEG 3350, 0.2 M MgCl_2_, 0.1 M Bicine pH 9.0, 1% v/v isopropanol
D187	monoclinic	15 mg∙mL^−1^	22% PEG 3350, 0.2 M Li_2_SO_4_, 0.1 M Bicine pH 9.0
C189	orthorhombic	16 mg∙mL^−1^	20% PEG 8000, 0.2 M Li_2_SO_4_, 0.1 M Bicine pH 9.0

### 2.5 X-ray data collection, crystal structure solution and refinement

Diffraction data were collected at the P13 EMBL beamline of the Petra III synchrotron at DESY, Hamburg, Germany. The diffraction images were processed with XDS (Kabsch, 2010). Crystal structures of the ReAV mutants were solved by molecular replacement using Phaser (McCoy et al., 2007) and the WT ReAV structure (PDB ID 7os5) as the starting model. Structures were refined with Phenix (Afonine et al., 2012) using anisotropic or TLS protocols (Winn et al., 2003). The electron density maps and solvent molecules were inspected in Coot (Emsley et al., 2010). All crystal structures were standardized in the crystallographic unit cell using the ACHESYM server (Kowiel et al., 2014). The statistics of data collection and structure refinement are summarized in Table 3. The structures were visualized using PyMOL (DeLano, 2002).

**TABLE 3:** Data collection and refinement statistics.

Structure	ReAV C135A	ReAV K138A	ReAV H139A	ReAV D187A	ReAV C189A
Data Collection					
Beamline	EMBL P13/Petra III, DESY Hamburg				
Wavelength (Å)	0.9763	0.9763	0.9196	0.8265	0.9763
Temperature (K)	100	100	100	100	100
Space group	*P*2_1_2_1_2_1_	*P*2_1_	*P*2_1_	*P*2_1_	*P*2_1_2_1_2_1_
Unit cell parameters (Å,°)	a = 78.06, b = 91.18, c = 106.05	a = 78.07, b = 91.39, c = 114.56, β = 96.89	a = 77.91, b = 91.47, c = 114.47, β = 96.98	a = 77.72, b = 91.26, c = 114.20, β = 96.95	a = 78.05, b = 91.29, c = 105.70
Oscillation range (°)	0.10	0.20	0.20	0.20	0.20
Number of images	3600	800	1800	1800	1800
Resolution range (Å)	78.06–1.71 (1.81–1.71)^a^	113.73–2.10 (2.23–2.10)	77.33–1.40 (1.49–1.40)	113.36–1.60 (1.69–1.60)	78.05–1.70 (1.81–1.70)
Reflections collected/unique	1003163/82131	278888/91345	2168042/309393	1465499/208980	1096881/83283
Completeness (%)	99.8 (99.1)	97.9 (98.8)	99.2 (98.1)	99.9 (99.5)	99.9 (99.7)
Multiplicity	12.2 (11.3)	3.1 (3.0)	7.0 (7.1)	7.0 (7.0)	13.2 (12.2)
Wilson B (Å^2^)	25.2	23.1	15.3	18.2	20.2
R_merge_ (%)	12.2 (188.3)	13.8 (85.8)	8.8 (132.2)	11.9 (128.6)	15.2 (164.9)
R_meas_ (%)	12.8 (197.0)	16.8 (104.6)	9.5 (142.7)	12.9 (138.8)	15.8 (172.1)
<I/σ>	11.89 (1.54)	7.72 (1.41)	12.68 (1.46)	12.66 (1.63)	14.95 (1.76)
CC_1/2_ (%)	99.8 (74.7)	99.2 (57.1)	99.9 (60.8)	99.9 (67.5)	99.9 (66.5)
Refinement					
Unique/test reflections	82042/1024	91119/999	309312/1544	208889/2087	83259/999
R_work_/R_free_ (%)	20.2/22.4	20.6/26.2	14.1/17.6	15.8/18.4	16.2/18.6
Protein chains in ASU	2	4	4	4	2
Matthews coeff. (Å^3^/Da)/solvent (%)	2.39/48.5	2.74/55.2	2.56/52.0	2.54/51.6	2.38/48.4
ADP model	TLS	TLS	Aniso	TLS	TLS
Protein/solvent/Zn atoms	5420/440/0	10521/722/0	10725/1479/4	10783/1534/4	5511/695/0
Rmsd bonds (Å)/angles(°)	0.010/1.07	0.010/1.07	0.009/1.07	0.010/1.08	0.010/1.06
Ramachandran plot (%) favored/allowed/outliers	97/3/0	97/3/0	97/3/0	97/3/0	98/2/0
PDB code	8rua	8rud	8rue	8ruf	8rug

^a^ Values in parentheses correspond to the highest resolution shell.

## 3 Results

### 3.1 Expression and purification of the generated ReAV mutants

Seven residues were subjected to site-directed point mutagenesis by substitution with alanine (Figures 1B, C). As mentioned above, the mutation sites were selected after careful examination of the WT ReAV crystal structure and comprehensive analysis of residue conservation profiles among all available bacterial orthologs. The correctness of the mutein sequences was confirmed by DNA sequencing. The two alanine mutants of cysteine residues involved in zinc coordination, C135A and C189A, were expressed in *E. coli* in substantial amounts in soluble form (∼40 mg∙L^−1^ of bacterial culture). A slightly higher expression yield (∼60 mg∙L^−1^) was obtained for the K138A mutant. The alanine substitution of the conserved His139 residue led to the formation of highly aggregated protein in inclusion bodies, although it was possible to recover ∼10 mg∙L^−1^ of the protein from cell lysate. The D187A mutant was produced in soluble form with satisfactory yield (∼25 mg∙L^−1^). Intriguingly, the mutation endows the protein with a pinkish color that may result from specific coordination of other (i.e., different from Zn^2+^) divalent metal cations. Mutants Y156A and C249A were expressed exclusively in insoluble form and the proteins could not be recovered from the cell lysate for activity tests.

### 3.2 Kinetic parameters of the active mutants, zinc profiles, and microcalorimetric titrations of the muteins with Zn^2+^

**FIGURE 2:**
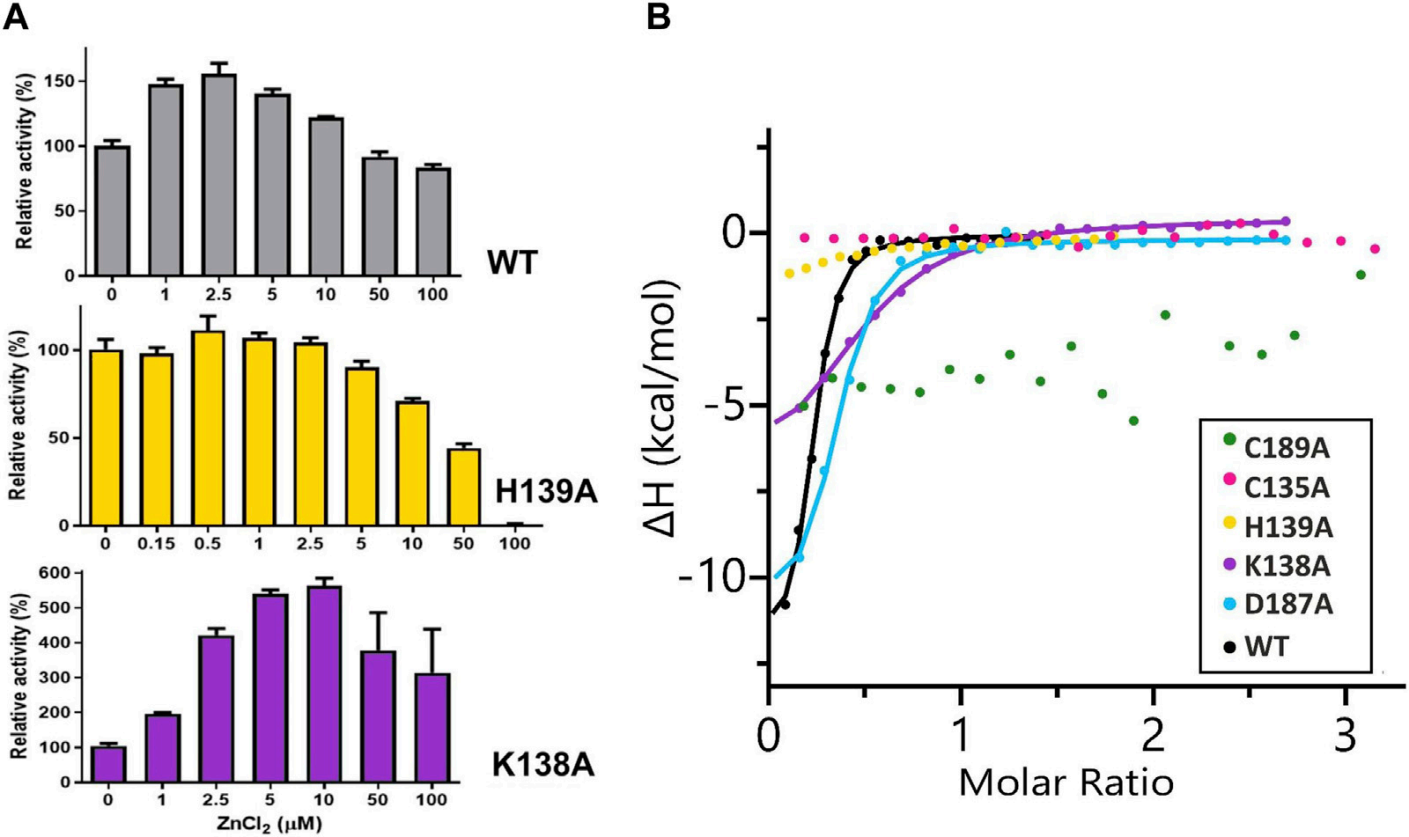
**(A)** Effect of different concentrations of ZnCl_2_ on WT ReAV and its K138A and H139A mutant activity, determined in 20 mM Tris-HCl buffer pH 9.0, by the Nessler reaction. The activity is expressed as a percentage of the activity of a control sample (not supplemented with zinc ions). The error bars are calculated from two separate experiments. **(B)** Representative binding isotherms from titrations of ReAV muteins with Zn^2+^ fitted with “one set of sites” model (the obtained K_D_ values are collected in Table 5).

No activity of the C135A, D187A, and C189A mutants was detectable either by the Nessler reaction or by ITC-MIM. The normalized zinc profile of the H139A mutant activity and the kinetic parameters of this mutant, with and without 500 nM Zn^2+^, are collected in Figure 2A and Table 4, together with normalized zinc profiles and kinetic parameters of the K138A mutant and WT protein, determined with and without their optimal Zn^2+^ concentration (Sliwiak et al., 2024). In order to additionally verify the state of occupancy of the zinc binding site, titrations of the muteins (not pretreated with any chelator) with Zn^2+^ were performed microcalorimetrically, leading to K_D_ determination for the K138A and D187A mutants only (Figure 2B; Table 5).

**TABLE 4:** Kinetic parameters of WT ReAV and its K138A and H139A mutants obtained by ITC-MIM titrations in 10 mM Tris-HCl buffer, without Zn^2+^ additive and in the presence of Zn^2+^ at the optimal 2.5 μM, 10 μM and 0.5 μM concentration, respectively. (±) Error estimates reflect agreement between two separate experiments.

ReAV variant	K_M_ [mM]	k_cat_ [s^−1^]	k_cat_/K_M_ [s^−1^·mM^−1^]
WT	2.7 ± 0.1	416 ± 3	154 ± 7
+Zn^2+^	2.1 ± 0.2	603 ± 24	294 ± 31
K138A	2.1 ± 0.1	4.8 ± 0.4	2.2 ± 0.1
+Zn^2+^	0.5 ± 0.1	6.5 ± 0.1	14 ± 3
H139A	5.6 ± 0.2	445 ± 20	80 ± 1
+Zn^2+^	5.1 ± 0.7	447 ± 85	87 ± 4

**TABLE 5:** Zn^2+^ affinity of WT ReAV and its muteins, together with their optimal Zn^2+^ concentration, their activity response to optimal and 100 μM Zn^2+^, and Zn^2+^ occupancy in the zinc-binding site in the crystal structures.

	WT	C135A	K138A	H139A	D187A	C189A
K_D__Zn^2+^_ [µM]	3.3 ± 0.8	n.b.	25.6 ± 2.0	n.d.	7.2 ± 2.0	n.b.
Zn^2+^ optimum [µM]	≥2.5, <5	-	≥10, <50	n.o.	-	-
specific activity with no Zn^2+^ [µmol·s^-1^·mg^-1^]	4.2 ± 0.2	0	0.030 ± 0.004	5.6 ± 0.4	0	0
effect of optimal Zn^2+^	55% activation	no effect^a^	467% activation	no effect^b^	no effect^a^	no effect^a^
effect of 100 µM Zn^2+^	7% inhibiton	-	214% activation	100% inhibition	-	-
Zn^2+^ occupancy in the crystal structure (PDB ID)	0.7 (7os5)	0 (8rua)	0 (8rud)	1 (8rue)	0.5 (8ruf)	0 (8rug)

n.b. - no binding detected, confirmed by structural data.

n.d. – no data due to full zinc saturation of the protein preparation, as indicated by structural data.

n.o. – no optimum detected; for concentrations ≤2.5 µM, Zn^2+^ causes no noticeable effect on the activity, above 5 µM of Zn^2+^ inhibition is observed.

- not measured.

^a^ Activity measured at 10 μM Zn^2+^.

^b^ Specific activity measured at 0.5 μM Zn^2+^ (inferred from the non-inhibiting concentration range of the H139A Zn^2+^ profile).

### 3.3 Description of the crystal structures of the mutant proteins

It was possible to crystallize five of the seven ReAV mutants. Single crystals of the muteins were grown at different crystallization conditions, and their quality varied depending on the mutation site. The investigated ReAV mutants crystallized in two space groups. The monoclinic structures (K138A, H139A, D187A) contain two ReAV dimers in the asymmetric unit (ASU), while the orthorhombic structures (C135A, C189A) have only one ReAV dimer per ASU. The crystals scattered X-rays to varying resolutions, even though they all had almost identical thin-plate morphology. Nevertheless, the final resolution of these datasets is very good, ranging from very high (1.40 Å) to moderate (2.10 Å).

#### 3.3.1 Mutant C135A

**FIGURE 3:**
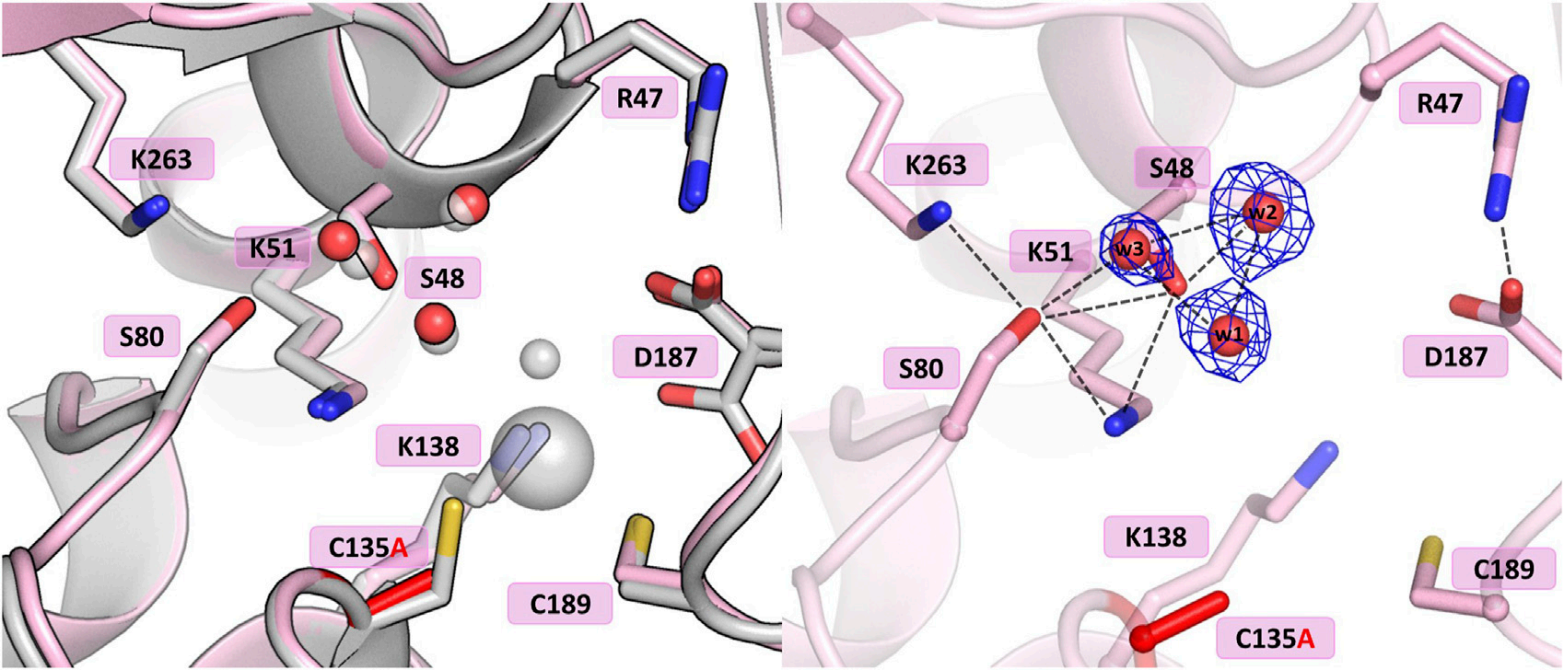
Structural changes in the ReAV C135A mutant. The left panel shows a superposition of the wild-type protein structure, along with the characteristic water molecules and metal cation (large sphere), used as a reference and shown in light gray, on chain A of the C135A mutant structure. The right panel shows a detailed view of the mutant active site (chain A) with 2Fo-Fc electron density (contour level 1.5σ) at the Ser48 water molecules. The mutation site is colored red. Hydrogen bonds are shown as black dashed lines. Water molecules are shown as small red spheres.

The crystal structure of this mutant revealed that the absence of Cys135 caused small structural rearrangements in the active site area, almost identical in the two subunits of the dimer, and triggered a collapse of the metal coordination site, since the mutant protein was unable to bind the Zn^2+^ cation. This disability was additionally confirmed by ITC affinity studies (Figure 2B; Table 5). The hydration pattern of Ser48 is nearly identical as in the WT protein, with three tightly bound water molecules in the close vicinity of the Ser48 hydroxyl group (Figure 3). These water molecules form strong hydrogen bonds with the Ser48 Oγ atom, with O…O distances in the range of 2.4–2.7 Å. In the close vicinity of these waters and Asp187, we observed a bound polyethylene glycol chain in subunit A or a glycerol molecule in subunit B, both located at the exit of the putative active site. The side chain of Ser48 is H-bonded to the Nζ atom of Lys51 and the Oγ atom of Ser80. The interaction between Ser48 and Ser80 stabilizes the protein structure (Loch et al., 2021) and is included in an extended network of H-bonds that fix the position of the Ser80 side chain and stabilize the non-planar configuration (Cα-C-N-Cα = 150°) of the Ala79-Ser80 peptide bond. The pattern of H-bonds at the two lysine residues from the Ser-Lys tandems, Lys51 and Lys263, is roughly the same as in the WT protein (Figure 1B). The Nζ atom of Lys51 is H-bonded to the Oγ atoms of Ser48 and Ser80, and also to the Oδ atom of Asn134 and the carbonyl O atom of Ala79. The side chain of Lys263 is H-bonded to the Oγ atom of Ser80, carbonyl oxygen of Leu264 and a water molecule. Only one conformer of Asp187 is visible in the electron density maps, with one of its Oδ atoms forming an H-bond to the Nε atom of Arg47. Interestingly, the side chain of Cys249 does not carry its typical chemical modification, which may be linked to the absence of the Zn^2+^ cation in the protein structure.

#### 3.3.2 Mutant K138A

**FIGURE 4:**
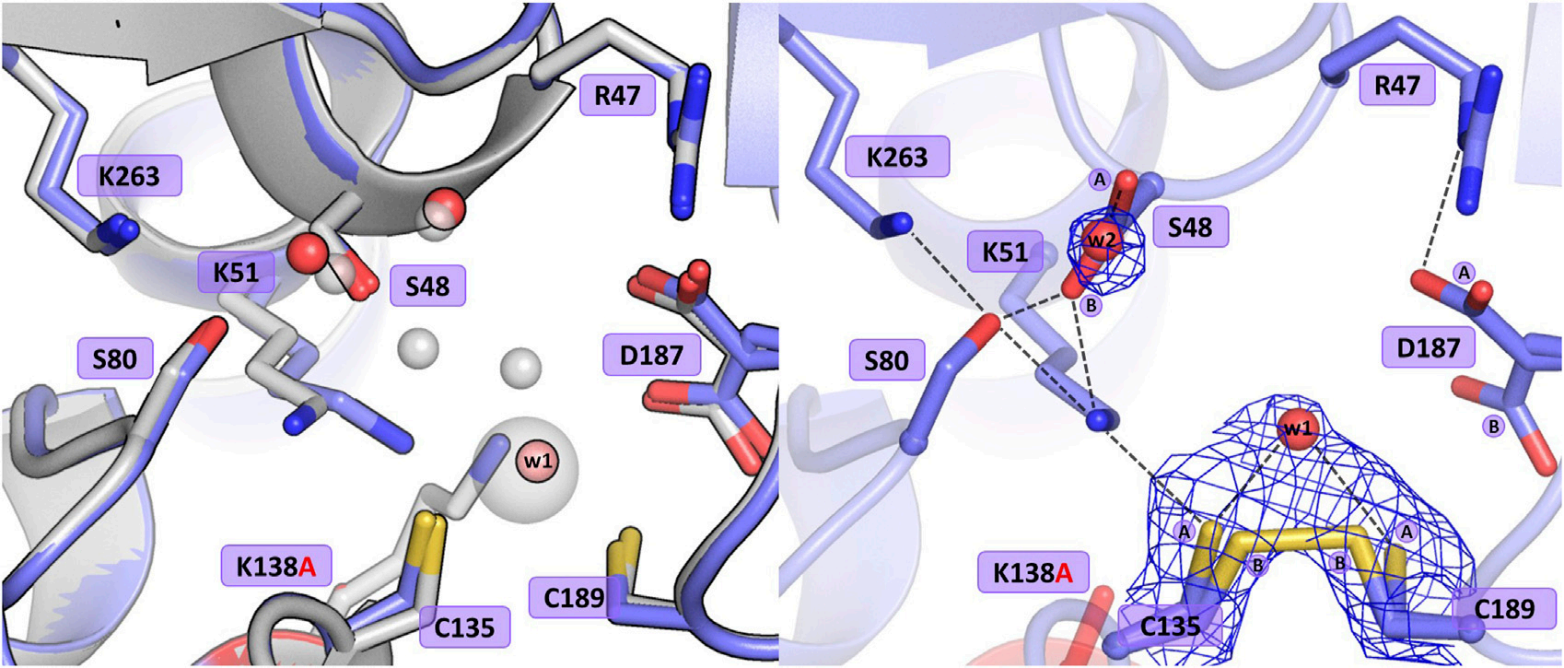
Structural changes in the ReAV K138A mutant. The left panel shows a superposition of the wild-type protein structure, along with the characteristic water molecules and metal cation (large sphere), used as a reference and shown in light gray, on chain A of the K138A mutant structure, with a water molecule (w1) bound by Cys135 and Cys189. The right panel shows a detailed view of the mutant active site (chain C) with 2Fo-Fc electron density (contour level 1.0σ) at the Cys135-Cys189 disulfide bridge and the water molecule w1 bound between the -SH groups of the cysteine residues in conformation A, as well as the Ser48 water molecule w2. The mutation site is colored red. The alternative conformations of Ser48, Cys135, Cys189, and Asp187 are indicated by circled capital letters A and B. Hydrogen bonds are shown as black dashed lines. Water molecules are shown as small red spheres.

The substitution of Lys138 with alanine caused significant structural rearrangements in the active site region. In the presence of the Lys138 mutation, the coordination site fell apart and is no longer accentuated by the metal cation. The absence of Lys138 affected the position and conformational states of Ser48 and Lys51 from the Ser48-Lys51 tandem, disrupting the H-bond network in the active site area, as well as the conformation of the cysteine residues from the zinc coordination sphere. In the four protein chains, the metal site was filled with a water molecule (w1) bound by the Sγ atoms of Cys135 and Cys189, and additionally, in protein chain C, alternative conformers of Cys135 and Cys189 form a direct Cys135-Cys189 disulfide bridge (Figure 4). In dimer AB, the side chain of Lys51 is rotated to a new position, with its Nζ atom H-bonded to the Sγ atom of Cys135 and the bound water molecule (chain A), and additionally to the Oγ atom of Ser48 in protein chain B. Interestingly, in subunit C of the CD dimer, the Lys51 side chain retained its typical conformation, while in chain D this residue adopts two conformations: one with the Nζ atom pointing to the Sγ atom of Cys135 and the Oγ atom of Ser48, and one facing the center of the substrate binding pocket. The Ser48 side chain has a slightly different position depending on the protein chain, without the characteristic pattern of three closely-spaced water molecules. In protein chains A and D, the side chain of Ser48 has the Oγ atom directed towards the Oγ atom of Ser80 as in the WT protein structure, and is also H-bonded to two water molecules (and additionally to the Nζ atom of Lys51 in protein chain D). A slightly different orientation of this residue can be observed in protein chain B, where the Ser48 side chain moves away from two neighboring water molecules and points to the Lys51 amine group. Interestingly, in subunit C, the side chain of Ser48 adopts two equivalent conformations, with only one adjacent water molecule. The side chain of Asp187 exists in two alternative conformational states in all four protein chains, with the major conformer rotated toward the side chain of Arg47.

The absence of Lys138 evidently impaired the mutant’s ability to bind the Zn^2+^ cation. Interestingly, the collapse of the metal binding site did not abolish the L-asparaginase activity of the mutant protein completely, as 1.5% of the activity (in terms of k_cat_/K_M_) was still observed (*vide supra*). Moreover, as shown by ITC affinity studies (Figure 2B; Table 5), the K138A mutant is still able to bind the zinc ion, albeit with lower affinity (∼26 μM) than the WT protein (∼3 μM) (Loch et al., 2021; Sliwiak et al., 2024). The low zinc binding affinity explains the absence of the zinc ion in the crystal structure. The side chain of Cys249 does not carry a chemical modification, which may be linked to the absence of the Zn^2+^ cation in the crystal structure, as in the case of the C135A mutant.

#### 3.3.3 Mutant H139A

**FIGURE 5:**
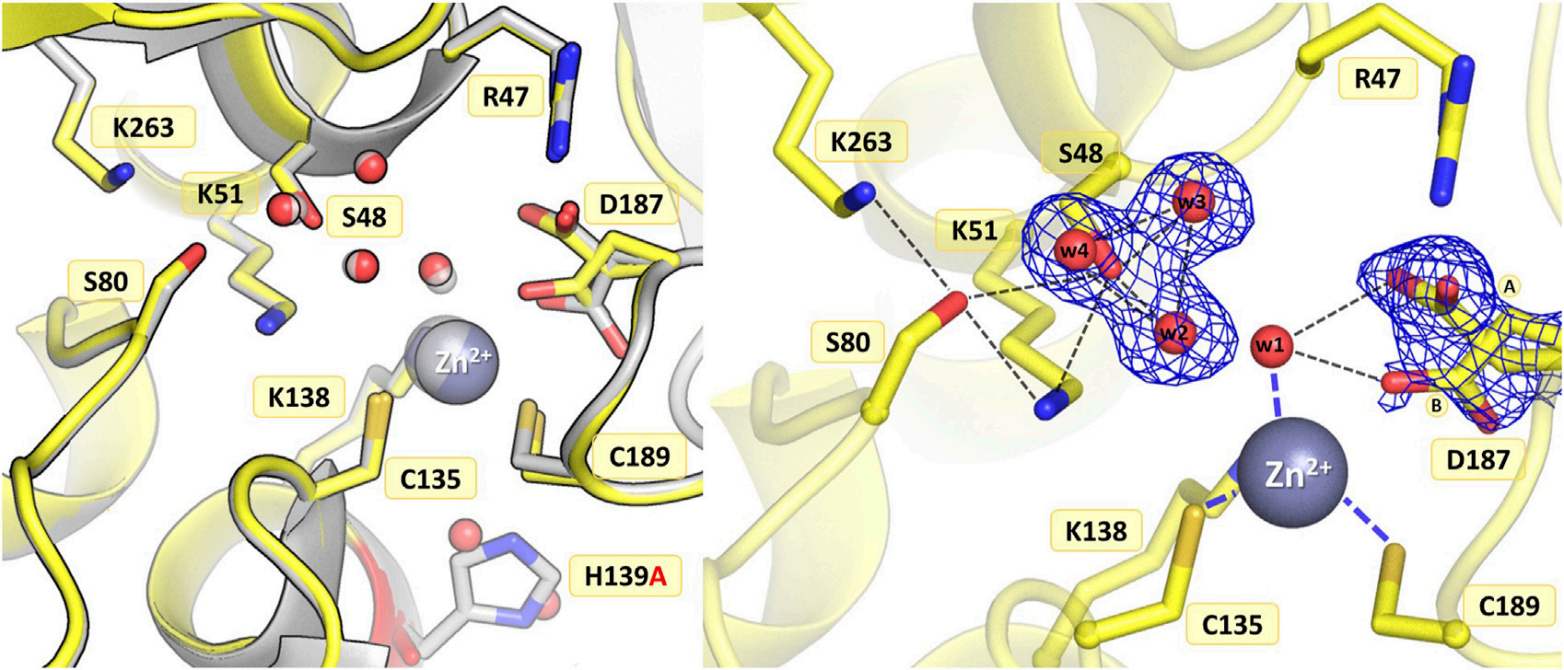
Structural changes in the ReAV H139A mutant. The left panel shows a superposition of the wild-type protein structure, along with the characteristic water molecules and metal cation (large sphere), used as a reference and shown in light gray, on chain B of the H139A mutant structure. The right panel shows a detailed view of the mutant active site (chain B) with 2Fo-Fc electron density (contour level 1.0σ) for the Ser48 water molecules and the alternative conformers of Asp187. The mutation site is colored red. The alternative conformations of Asp187 are indicated by circled capital letters A and B. Hydrogen bonds are shown as black dashed lines. Water molecules are shown as small red spheres.

The introduction of alanine at position 139 resulted in only minor atomic shifts and structural rearrangements in the active site area, almost identical in the four protein chains. The substitution of His139 by alanine does not affect the conformation of the neighboring residues: just two water molecules, marking the sites of the two N atoms of the imidazole ring, fill the available space left by the histidine side chain, maintaining the original pattern of hydrogen bonds in the active site area (Figure 5). The fully occupied Zn^2+^ binding site is conserved in the mutant structure, as are the catalytic Ser-Lys tandems. In the close vicinity of the Ser48 hydroxyl, high difference electron density peaks were detected, previously also observed in the WT protein structure and interpreted as three closely spaced water molecules (Loch et al., 2021). Therefore, a water triad was modeled forming very strong H-bonds with the Ser48 Oγ atom, with O…O distances of 2.3–2.4 Å. The crystal structure of ReAV H139A shows alternative conformations of Asp187 in protein chain B. The major rotamer of Asp187 (refined occupancy 0.7) is rotated toward the side chain of Arg47, forming an H-bond to its Nε atom, as well as to the water molecule from the zinc coordination sphere and the Oγ atom of Thr341. The minor rotamer forms H-bonds to the main chain N atom of Leu191 and two water molecules, one of which belongs to the metal coordination sphere. The pattern of H-bonds at the two lysine residues from the Ser-Lys tandems, Lys51 and Lys263, is roughly the same as in the WT protein (Figure 1B). Interestingly, the side chain of Cys249 is not oxidized, in contrast to what was observed previously when the zinc cation was present in the WT protein structure (Loch et al., 2021).

#### 3.3.4 Mutant D187A

**FIGURE 6:**
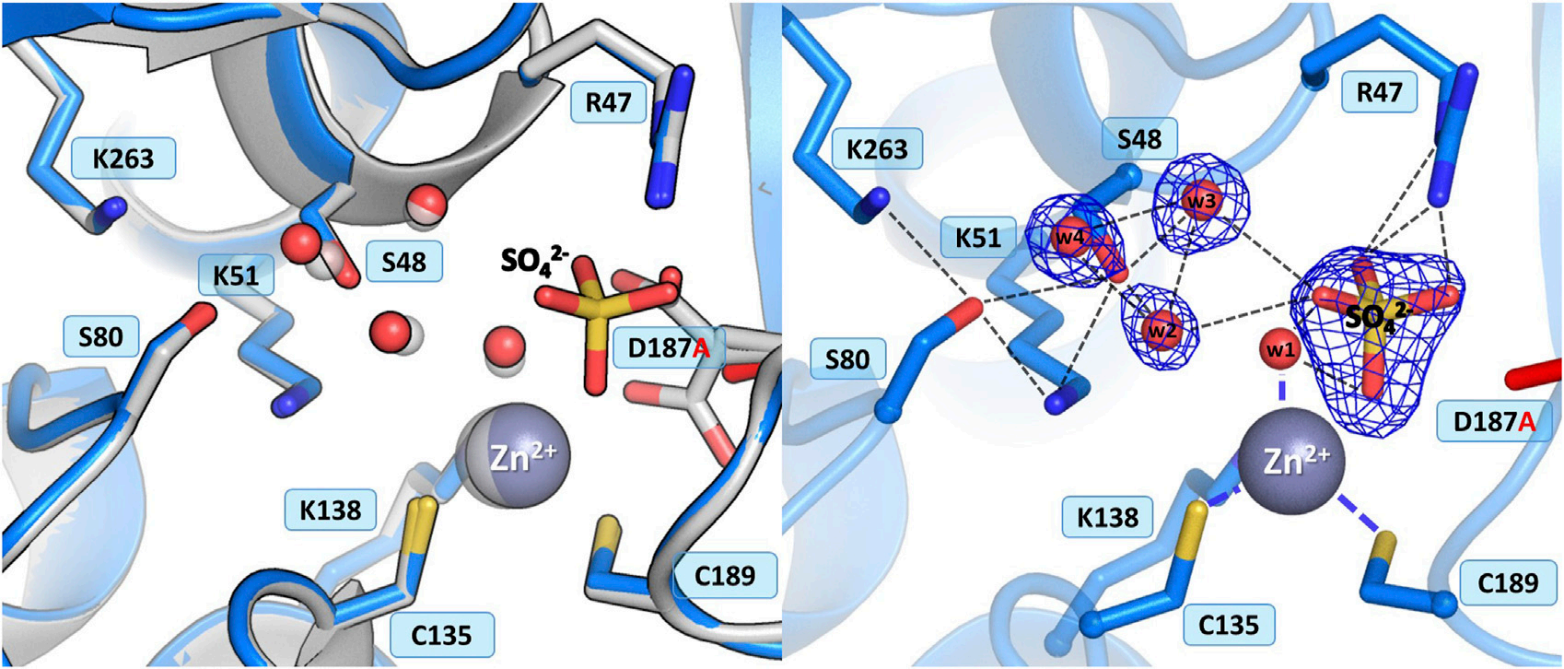
Structural changes in the ReAV D187A mutant. The left panel shows a superposition of the wild-type protein structure, along with the characteristic water molecules and metal cation (large sphere), used as a reference and shown in light gray, on chain A of the D187A mutant structure. The right panel shows a detailed view of the mutant active site (chain A) with 2Fo-Fc electron density (contour level 1.5σ) at the Ser48 water molecules and sulfate ion. The mutation site is colored red. Hydrogen bonds are shown as black dashed lines. Water molecules are shown as small red spheres.

In the WT protein, Asp187 adopts alternative conformations, with its side chain rotated toward the water molecule from the zinc coordination sphere or pointing to the N atoms of Ala189 and Asn190, thus maintaining the network of hydrogen bond connections within the active site region and controlling access to the metal cation (Figure 1B). The ReAV D187A crystal structure revealed that the absence of Asp187 induced only small local conformational changes and atomic shifts, and these changes are strictly equivalent in the two dimers. The zinc cation is only partially present at the metal binding site, with a refined occupancy of 0.5, as is the w1 water molecule from the metal coordination sphere. Moreover, substitution of Asp187 with alanine affected the metal environment, since a sulfate ion occurs right next to the strongly bound water molecule from the zinc coordination sphere (Figure 6). The sulfate ion fills a central position in the active site cleft, possibly preventing the enzyme from accepting a substrate molecule. The negatively charged SO_4_^2-^ anion forms a salt bridge with the protonated and positively charged side chain of Arg47. It is also H-bonded to the main chain N atoms of Gly188 and Cys189, and surrounded by four water molecules, two of which belong to the Ser48 hydration sphere. The hydration pattern of Ser48 is nearly identical as in the WT protein, with three closely-spaced water molecules in the vicinity of the Ser48 hydroxyl group. These water molecules are strongly H-bonded to the Oγ atom of Ser48, with O…O distances in the range of 2.3–2.7 Å. The pattern of H-bonds at the two lysine residues from the Ser-Lys tandems, Lys51 and Lys263, is practically the same as in the WT protein. Surprisingly, the side chain of Cys249 is not oxidized to sulfenic acid, which may be related to a change in the zinc environment.

#### 3.3.5 Mutant C189A

**FIGURE 7:**
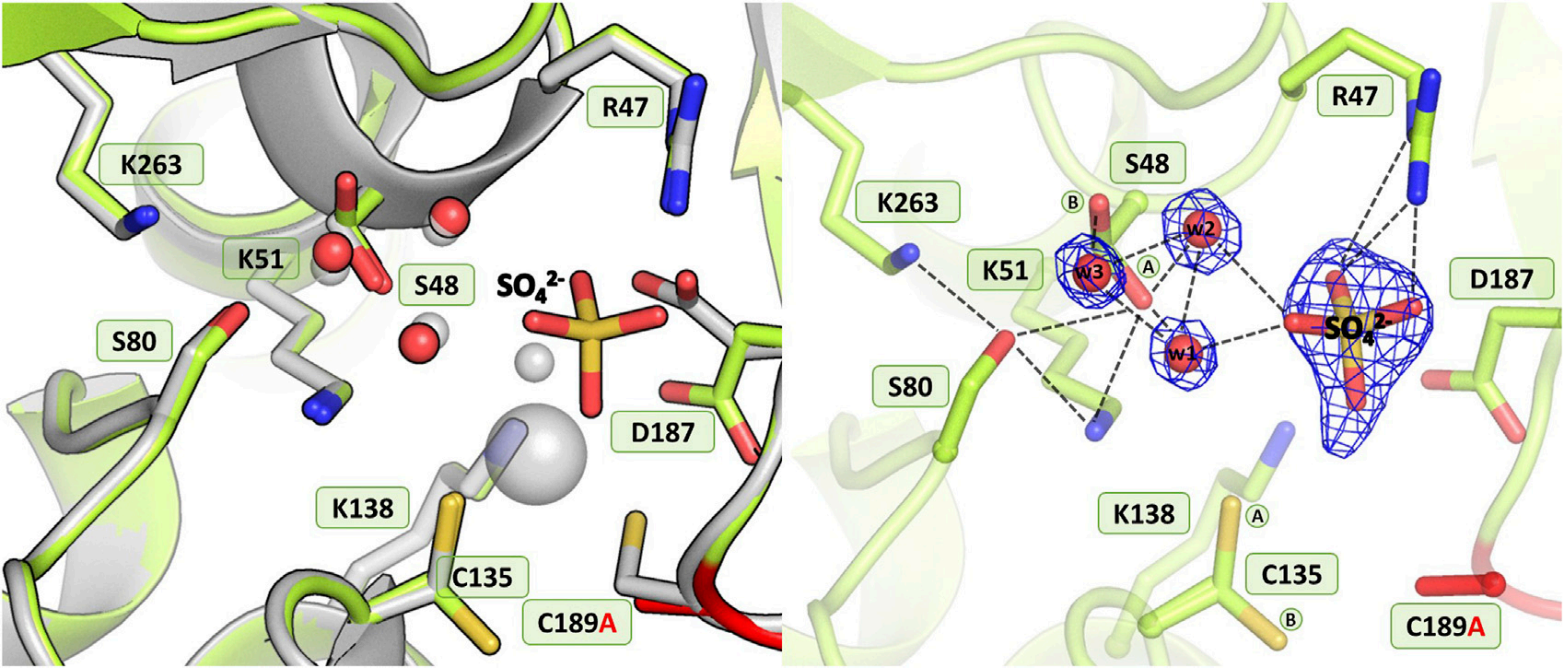
Structural changes in the ReAV C189A mutant. The left panel shows a superposition of the wild-type protein structure, along with the characteristic water molecules and metal cation (large sphere), used as a reference and shown in light gray, on chain B of the C189A mutant structure. The right panel shows a detailed view of the mutant active site (chain B) with 2Fo-Fc electron density (contour level 1.5σ) at the Ser48 water molecules and sulfate ion. The mutation site is colored red. The alternative conformations of Ser48 and Cys135 are indicated by circled capital letters A and B. Hydrogen bonds are shown as black dashed lines. Water molecules are shown as small red spheres.

As in the structure with the C135A mutation, the absence of Cys189 triggered a collapse of the metal coordination site and the C189A mutant protein is unable to bind the Zn^2+^ cation (Figure 7), as also corroborated by microcalorimetric titration of this protein with zinc (Figure 2B; Table 5). The mutation induced slight atomic shifts and structural rearrangements in the active site area, but these changes are not strictly equivalent in the two subunits of the dimer. Alternative conformers of Cys135 were found in protein chain B, where the major rotamer with refined occupancy of 0.8 retained its typical conformation and points to the Nζ atom of Lys51, while the minor rotamer is rotated toward the Oγ atom of Ser133. The hydration pattern of Ser48 is similar as in the WT protein, with a water triad centered around the Ser48 hydroxyl group. However in subunit B, the Ser48 side chain exists in two conformational states, with the major rotamer, with refined occupancy of 0.7, directed toward the Ser80 hydroxyl group, and the lower-occupancy rotamer pointing to the carbonyl O atom of Leu264. The pattern of H-bonds at the two lysine residues, Lys51 and Lys263, is practically the same as in the WT protein (Figure 1B). Only one conformer of Asp187 is visible in the electron density maps, but intriguingly, its side chain points to the main chain N atoms of Ala189, Asn190 and Leu191, and not toward the side chain of Arg47, as observed in the previously described C135A and H139A mutant structures. This drastic change in the position of Asp187 is undoubtedly caused by the presence of a sulfate ion (at 0.8 occupancy) that replaced Asp187 in the interaction with Arg47, with which it forms a salt bridge. At pH 9.0, the side chain of Asp187 is deprotonated and moves away from the highly negative SO_4_^2-^ anion. Apart from the interaction with Arg47, the sulfate ion forms H-bonds to the N atom of Gly188 and to four water molecules, two of which belong to the hydration sphere of Ser48. The position of the sulfate ion is slightly shifted compared to its position in the D187A mutant structure, where the sulfate ion is bound right next to the water from the metal coordination sphere. As in the case of the C135A and K138A variants lacking the metal cation, the mutation affected the oxidative state of Cys249, which is not oxidized.

## 4 Discussion

### 4.1 Insights from massive analyses of bacterial genomes

A pan-genomic analysis of bacterial sequences performed to identify orthologs of ReAV genes across all taxonomic units in the Bacteria domain (Zielezinski et al., 2022), reported that type V L-asparaginase (exemplified by ReAV) is present in over 1500 species, mostly in the families of *Cyanobiaceae* and *Burkholderiaceae*, and only rarely occurs in the *Rhizobiaceae* family (23 species), in contrast to the type IV sequence (exemplified by ReAIV), which is indeed characteristic of *Rhizobia*. Since the ReAV enzyme does not possess orthologs in closely related bacteria but has a high sequence similarity ortholog in the distantly related *Burkholderia ubunensis*, it was concluded that *Rhiozbium etli* received the ReAV gene from *Burkholderia* through an HGT event. Mapping the level of residue conservation within the orthologous family on ReAV structure identified the most conserved regions; all located in the active site area and its close vicinity. No favorable substitutions of the catalytic site residues could be observed, with a level of residue conservation of 99% for Ser48, Lys51, Ser80, Cys135, Cys189, Lys263, and 97% for Lys138. The created sequence profile also indicated a few neighboring residues that are preserved in 99% of the orthologs and do not have preferable replacements, namely Arg47, His139, Tyr156, and Asp187. Such high conservation of the active site residues can presumably be correlated with the enzymatic properties of ReAV and similar proteins. Cys249 is the only residue from the general metal environment with a low level of conservation (30%), and except for the most frequent Cys, Asp can also be found at this position. The selected residues became the subject of our mutational studies aimed at confirming their functional role and elucidating a possible mechanism of action.

### 4.2 Exploring the importance and properties of the selected amino acid residues

Two most conserved patterns of the ReAV sequence were selected for mutagenesis based on extensive analyses of the available bacterial genomes and on comparisons with available structural homologs. Those patterns could be defined structurally as follows. Pattern I, residues involved directly in zinc coordination (Cys135, Lys138, Cys189), as well as residues in the close vicinity of the metal binding site (His139, Tyr156, Asp187, Cys249), and Pattern II, residues forming the two Ser-Lys tandems and their neighborhood (Arg47, Ser48, Lys51 Ser80, Lys263). The focus of this paper is on directed mutagenesis of Pattern I. Mutagenesis of Pattern II will be described in a forthcoming paper.

Apart from metal binding, Cys135, Lys138, and Cys189 in the WT structure also participate in a network of H-bonds in the active site area (Figure 1B). The positions of Cys135 and Cys189 are stabilized by H-bonds to the side chains of Lys51 and His139, respectively. The Lys138 side chain interacts with two water molecules that mediate H-bonding with the side chain of Gln54 (Figure 1C). Tyr156 is mostly surrounded by hydrophobic residues and its position is stabilized by H-bonds to the side chain of His139 and the main chain N atom of Arg106. The side chain of Tyr156 fills the empty space between these two residues and is likely a factor stabilizing the protein fold (Figure 1C). The side chain of Asp187 is rotated toward the water molecule in the zinc coordination sphere, but may adopt an alternative conformation, pointing toward Cys189. Asp187 maintains the pattern of hydrogen bonds in the active site region, at the same time guarding access to the metal cation (Figures 1B, C). A relatively distant Cys249 residue, carrying a posttranslational modification, namely oxidation to sulfenic acid, is indirectly connected by H-bonds to the side chain of Cys135 and potentially protects the metal binding site from oxidative damage (Loch et al., 2021). Analysis of the level of amino acid frequencies in known bacterial orthologs revealed that, with the exception of the oxidizable Cys249, all other residues forming Pattern I are strictly conserved, with a very low probability of residue substitutions. In agreement with this level of conservation, the experiments presented in this work confirm that all the residues selected as Pattern I are undoubtedly crucial for enzymatic activity, with the possible exception of His139.

### 4.3 Comparison of the kinetic parameters and response to Zn^2+^ of two ReAV mutants

The K138A and H139A mutants are the only active enzymes among the studied muteins. The total knockout of the activity (even in the presence of Zn^2+^) after substitution of the C135, D187, and C189 residues with alanine indicates their key role in the mechanism of the catalytic reaction. The K138A mutation, which impairs but does not abolish the zinc binding ability, slows down the enzyme significantly (k_cat_ = 4.8 s^−1^ vs. 416 s^−1^ for WT) but also slightly improves its substrate specificity (K_M_ = 2.2 mM vs. 2.7 mM for WT) (Table 4). Interestingly, the H139A mutant matches the WT form in k_cat_ (445 s^−1^ vs. 416 s^−1^), but its substrate specificity is reduced more than twice (5.6 mM vs. 2.7 mM). We already demonstrated the boosting effect of low and optimal zinc concentration on substrate specificity (which in itself indicates a role of Zn^2+^ cations in substrate recognition) and turnover number of rhizobial asparaginases. The optimal zinc concentration for WT ReAV activity appeared to be 2.5 μM (Figure 2A), which is close to the K_D_ of zinc binding (∼3 μM) by this protein. This optimal zinc concentration boosted the ReAV activity by more than 50% (Sliwiak et al., 2024). We could also observe that crippling the zinc coordination by the K138A mutation shifted this Zn^2+^ optimum to 10 μM, and eliminated the slight inhibitory effect of 100 μM zinc concentration observed for WT ReAV (100 μM Zn^2+^ is still strongly stimulative for K138A). Moreover, the mutant activity is boosted by optimal Zn^2+^ in this case by as much as 500% (Figure 2A), although the enzyme efficiency, even in the presence of optimal Zn^2+^, is 20 times lower than in the case of the WT protein. 10 μM Zn^2+^ greatly improves the K_M_ of ReAV K138A from 2.2 to 0.5 mM, making this mutant an excellent candidate for substrate co-crystallization experiments, and indeed for further optimization toward antileukemic properties. The elevated optimal Zn^2+^ concentration in the case of the K138A mutant, the lack of inhibitory effect of 100 μM Zn^2+^ (Figure 2A), and the dramatically reduced activity (Table 4) of this mutant, could all be the effect of its lower Zn^2+^ affinity compared to the WT protein (Figure 2B; Table 5).

Interestingly, the H139A mutant shows a completely different response to the presence of zinc ions (Figure 2A; Tables 4, 5), as (i) we did not observe a clear optimum of Zn^2+^ concentration even in the nanomolar range, and the activity of this mutant at low Zn^2+^ concentration range (0–2.5 μM) is within the margin of error comparable to that of the unsupplemented protein; (ii) Zn^2+^ at non-inhibiting concentration range has no significant impact on the kinetic parameters; (iii) 100 μM of Zn^2+^ turns off the enzyme activity completely. We interpret these observations as indicating a very strong zinc binding by the H139A ReAV mutant, as in Sliwiak et al. (2024) we observed that the activity of the other *R. etli* isoform ReAIV (which binds Zn^2+^ thrice as strongly as ReAV) is over three times more susceptible to inhibition by 100 μM Zn^2+^ (24% vs. 7%) than ReAV. We also could not titrate the H139A mutant with Zn^2+^ because, as revealed by the crystal structure, it is already fully saturated with zinc. Thus the absence of zinc binding by the H139A mutein in ITC assays has a completely different origin than in the case of the C135A and C189A mutants, which are unable to coordinate Zn^2+^ at all. Interestingly, the D187A mutant, which binds Zn^2+^ nearly as well as the WT protein (K_D_ 7.2 vs. 3.3 μM, Figure 2B; Table 5) is not active even in the presence of Zn^2+^ ions, meaning that its inactivity is caused by factors other than zinc binding capacity.

### 4.4 Insights from structural analysis of the created ReAV muteins

In this work, we reported five crystal structures of ReAV site-directed mutants: two orthorhombic structures (C135A and C189A) with a dimer in the ASU, and three monoclinic structures (K138A, H139A, and D187A) with two dimers per ASU. Depending on the mutation introduced, these structures present different conformational states of Ser48, Lys51, Cys135, Asp187 and Cys189, as well as some conformational perturbations and atomic shifts of residues within the active site area. The study reveals that modification of the highly conserved residues in the vicinity of the Zn^2+^ cation does not always abolish the L-asparaginase activity.

It is important to emphasize that elimination of any of the cysteine residues involved in zinc coordination (Cys135, Cys189) rendered the mutant proteins unable to bind the metal cation, as shown by the structural and affinity studies. The C135A and C189A mutants also lost the ability to hydrolyze L-asparagine. The K138A substitution, however, only decreased the zinc binding affinity compared to WT, and at the same time reduced the mutein ability to hydrolyze L-asparagine nearly 40-fold. Both observations, i.e., complete loss of enzymatic activity after elimination of zinc binding (C135A and C189A), and simultaneous reduction of enzymatic activity and zinc binding affinity (K138A) demonstrate that the zinc cation could play an essential role in the catalytic process. Since the K138A mutant binds Zn^2+^ weakly, this mutein could lose this ion during the purification process. This could explain why zinc was not detected in the crystallographic electron density maps. Moreover, the crystal structure of ReAV K138A shows that in the absence of the Zn^2+^ cation, a structurally equivalent Cys135-Cys189 disulfide bridge can be formed, stabilizing the active site region. Such a weak Zn^2+^ binding could also be deduced from the zinc profile of the K138A mutant (Figure 2A). 100 μM Zn^2+^ has no inhibitory effect on this mutein, in contrast to the WT protein, and the optimal Zn^2+^ concentration (10 μM) is four times higher than for the WT protein. Also, the zinc profile of K138A shows that the activity of this mutein increases dramatically (by 500% instead of 50% for WT, Figure 2A) when supplemented with zinc ions. Because of its weak zinc binding, the K138A mutant should be kept at elevated Zn^2+^ concentration for relevant kinetic and biophysical assays. The C135A and C189A mutants are not active at all, even in presence of Zn^2+^, which is consistent with their inability to bind zinc in our ITC studies.

The structures of the C135A and C189A mutants demonstrate that alanine substitution of the cysteine residues involved in zinc coordination led to collapse of the metal binding site and abrogation of the L-asparaginase activity. The mutations also affected the hydrogen-bond network in the active site region and the altered pattern does not seem to be sufficient for the correct positioning of the substrate molecule. It would, therefore, appear that proper substrate binding and concomitant catalytic activity require the precise, native network of hydrogen bonds in the active site. The Ser48 hydroxyl group from one of the Ser-Lys tandems is surrounded by three water molecules, as in the WT protein structure. Moreover, mutants C135A and C189A differ in the position of Asp187. In the ReAV C135A structure, the side chain of Asp187 is rotated toward the side chain of Arg47, whilst in the C189A structure the presence of a strongly negative sulfate anion right in the catalytic center forced the anionic side chain of Asp187 toward the N atoms of Ala189, Asn190, and Leu191.

Despite the mutation of the highly conserved His139, the H139A mutein retained the L-asparaginase activity. Furthermore, the very high quality of the electron density maps showed the familiar hydration pattern of the Ser48 residue, first observed in WT ReAV, where three water molecules form extremely strong H-bonds with the Oγ atom of Ser48, as well as within their triangle. These data are in good agreement with previous suggestions that Ser48 might act as the primary nucleophile during the hydrolysis reaction.

The alanine substitution of Asp187, which gates access to the metal cation, affected the metal environment and allowed the binding of a sulfate anion right in the catalytic center. Although Ser48 remained hydrated in the D187A mutant, the mutation completely abolished the enzymatic activity, despite the fact that this mutein can still bind zinc (K_D_ of 7.2 μM, Table 5). This observation may be correlated with the presence of the sulfate ion in the active site, which would hinder the acceptance of a genuine substrate molecule. The crystal structure of the D187A mutant clearly showed that the metal binding site is only partially occupied by Zn^2+^ cations. Interestingly, the Asp187 mutation resulted in the production of a pinkish protein. We hypothesize that with altered metal binding properties, the coordination site of the D187A mutant could become specific for other transition metal ions, which could be bound during protein bacterial expression or purification on Ni^2+^ resin.

In all structures presented in this paper, the side chain of Cys249 is not oxidized, in contrast to what is typically observed. It was hypothesized that this oxidative modification may play a role in protecting the zinc binding site against ROS (Loch et al., 2021). When the zinc cation is not present in the crystal structure due to the elimination of residues directly involved in its coordination, i.e., Cys135, Lys138 or Cys189, the lack of Cys249 oxidative modification may be linked to the absence of the metal ion. A similar situation could be observed in the structure of the D187A mutant, where a sulfate anion is present right next to the water from the metal coordination sphere; therefore, the lack of chemical modification of the Cys249 side chain may be associated with a change in the zinc environment. Intriguingly, the side chain of Cys249 did not undergo oxidative modification in the H139A mutant structure either, in contrast to what had been observed previously when the zinc cation was present in the WT protein structure (Loch et al., 2021).

### 4.5 Insights from ReAV muteins for which structural analysis was impossible

Two of the designed mutants, Y156A and C249A, could not be properly expressed, most likely because of folding problems. However, the negative results may also be the source of useful insights. In the WT ReAV structure, the bulky aromatic ring of Tyr156 occupies a substantial volume of the hydrophobic core of the protein and its hydroxyl group forms H-bonds to the side chain of His139 and the main chain of Arg106 (Figure 1C), and these connections seem to be crucial for the stabilization of the protein core and for proper folding. Thus, without Tyr156 the protein core would not be properly formed.

The side chain of Cys249 (in both, oxidized and non-oxidized form) is involved in a network of water-mediated H-bonds to neighboring residues, e.g., to the main chain of Leu264 and Val242, or the side chains of Tyr304 and Arg247, reaching as far as the entire active site (Figure 1B). Thus, the absence of cysteine at position 249 would perturb an extended pattern of H-bond connections in the most sensitive part of the protein architecture, leading to destabilization of the entire ReAV fold. We can thus speculate that without the Cys249 lynchpin (or similar residue) the protein would not be able to fold properly.

## 5 Conclusion and outlook

The inducible Class 3 L-asparaginase ReAV from the nitrogen-fixing bacterium *Rhizobium etli*, is potentially interesting as a novel antileukemic candidate. Since it shows no sequence or structural similarity to known enzymes with this activity, it may offer completely new avenues of approach to enzyme engineering. Moreover, as a protein unrelated to the clinically used enzymes, it would evade the severe immunological side effects accompanying the current therapeutic protocols. The evolutionary origin of Class 3 L-asparaginases remains an intriguing question. Despite low sequence similarity, the ReAV enzyme shares a similar fold with glutaminases and the related serine β-lactamases. Moreover, from a recent pan-genomic analysis of the distribution of ReAV orthologs within the bacterial kingdom (Zielezinski et al., 2022), we can conclude that the ReAV enzyme shows the highest sequence similarity to proteins found in *Burkholderia* species, and the presence of ReAV in *Rhizobium etli* may be the result of an HGT event from *Burkholderia*. Mapping the level of residue conservation within the orthologous family on the ReAV structure allowed us to identify the most conserved regions, located in the putative active site area. We assumed that such high conservation of residues should be associated with the enzymatic mechanism of ReAV. Our mutational studies indeed showed that modification of the highly conserved residues, i.e., Cys135, Asp187, and Cys189, completely abolished the L-asparaginase activity. Interestingly, alanine substitution of the highly conserved Lys138 from the zinc coordination sphere rendered the mutant protein partially active. Because prevention of zinc binding (C135A, C189A) turns the activity off, and reduced zinc affinity (K138A) is correlated with a significant reduction of the enzymatic activity, we postulate that Zn^2+^ ions may play a role in ReAV catalysis. We are planning to use the K138A mutant for large-scale co-crystallization experiments with L-Asn and L-Asp in order to generate a substrate/product complex. So far, our kinetic and crystallographic results have provided many useful hints about the catalytic mechanism of the enzyme, but we are not there yet to decipher it completely.

## Data Availability

The datasets presented in this study can be found in two online repositories. Atomic coordinates of the models and structure factors are in the Protein Data Bank (PDB, http://www.wwpdb.org/): https://doi.org/10.2210/pdb8RUA/pdb (8rua), https://doi.org/10.2210/pdb8RUD/pdb (8rud), https://doi.org/10.2210/pdb8RUE/pdb (8rue), https://doi.org/10.2210/pdb8RUF/pdb (8ruf), https://doi.org/10.2210/pdb8RUG/pdb (8rug); and raw diffraction data are in Macromolecular Xtallography Raw Data Repository (MX-RDR, https://mxrdr.icm.edu.pl): https://doi.org/10.18150/KTMCG7 (8rua), https://doi.org/10.18150/RBG2F9 (8rud), https://doi.org/10.18150/F9YMXN (8rue), https://doi.org/10.18150/BWIJSJ (8ruf), https://doi.org/10.18150/BJQWOX (8rug).

## Author’s note

The PDB validation report for the structure of the C135A mutant (PDB code: 8rua) shows an exceptionally high value (35.3%) of the Real-Space R-value Z-score (RSRZ) describing the fit of the model to the electron density map. A detailed visual inspection of the electron density map and the model proves that the residues from the list of RSRZ outliers fit the electron density very well, similarly to other presented structures, where the RSRZ has values of less than 1%. According to information on the official wwPDB website (https://www.wwpdb.org/validation/FAQs#RSRZ), such a situation most likely indicates that the REFMAC program (used internally by the PDB deposition server) did not calculate the maps correctly. The PDB team apologizes and states that they are working on alternative procedures to improve the reliability of map fitting validation.
